# Updates on Ecology and Life Cycle of *Sulcascaris sulcata* (Nematoda: Anisakidae) in Mediterranean Grounds: Molecular Identification of Larvae Infecting Edible Scallops

**DOI:** 10.3389/fvets.2020.00064

**Published:** 2020-02-14

**Authors:** Federica Marcer, Federica Tosi, Giovanni Franzo, Alessia Vetri, Silvia Ravagnan, Mario Santoro, Erica Marchiori

**Affiliations:** ^1^Department of Animal Medicine, Production and Health, Padova University, Legnaro, Italy; ^2^National Reference Center for Fish, Molluscs and Crustacean Diseases, Istituto Zooprofilattico Sperimentale delle Venezie, Legnaro, Italy; ^3^Parasitology Laboratory, Istituto Zooprofilattico Sperimentale delle Venezie, Legnaro, Italy; ^4^Department of Integrative Marine Ecology, Stazione Zoologica Anton Dohrn, Naples, Italy

**Keywords:** *Sulcascaris sulcata*, life cycle, Pectinidae, molluscs, Anisakidae, Mediterranean Sea, *Caretta caretta*

## Abstract

*Sulcascaris sulcata* is a nematode parasite of sea turtles, widespread in neritic foraging grounds with variable prevalence, reaching 30% in loggerhead turtles *Caretta caretta* feeding in northern Adriatic Sea. Ulcerative gastritis associated to high intensity of infection is reported in this host species. The life cycle of *S. sulcata* has been elucidated in Australian and American waters, demonstrating the ability of the species of infecting a wide range of intermediate hosts, represented by bivalve and gastropod molluscs. During regular sanitary inspections, nematode larvae were found within the adductor muscle of *Pecten jacobeus* and *Aequipecten opercularis* collected from the Northern Adriatic Sea. Morphological and molecular analyses were performed for the identification of larvae, and molecular data were obtained from adult *S. sulcata* as well. Analysis of the sequences of ITS fragment, and mitochondrial genes *cox*1 and *cox*2, revealed high genetic similarity among all the samples, and no geographical clustering was observed between adult parasites collected in Adriatic and Tyrrhenian Seas. A common allele pool was detected also between the two developmental stages, included larvae from *Pecten* and *Aequipecten*. The comparison with other members of the family Anisakidae demonstrated that *S. sulcata* formed a clear monophyletic cluster. This study reports the first identification of intermediate hosts for *S. sulcata* within the Mediterranean Sea. Infection in edible scallops justifies the exclusion of the product from the market and zoonotic potential of larvae of this anisakid nematode are yet to be completely excluded. Fidelity of sea turtles to selected foraging grounds, such as the Northern Adriatic shelf, warrants the life cycle of *S. sulcata* to perpetrate in the area; at the same time, long distance migrations of individuals justify the dispersal of infecting elements over the Mediterranean basin, regardless of turtles' origin.

## Introduction

*Sulcascaris sulcata* (Rudolphi, 1819) (Nematoda: Anisakidae) is a nematode parasite of sea turtles, with a wide geographical distribution in marine ecosystems (1–8). Adult parasites have been described within the gastric lumen of loggerhead (*Caretta caretta*), Kemp's ridley (*Lepidochelys kempii*) and green turtles (*Chelonia mydas*), in association with variable degrees of ulcerative gastritis (3, 9, 10). Infection by *S. sulcata* has been extensively reported within the Mediterranean basin, in particular from neritic grounds, such as the Adriatic Sea (5, 10–13), the African shelf (1) and coastal waters off Campania region (6). Higher prevalence and abundance of *S. sulcata* in larger turtles, feeding in inshore waters is expected, due to the higher probability of ingestion of mollusc intermediate hosts (13). Several species of bivalves have been reported to carry infectious larvae of *S. sulcata* in waters off America and Australia (9, 14–18), including species of the family Pectinidae. Molluscs of this family are part of loggerhead turtle diet also in the Adriatic feeding grounds (19) but no intermediate hosts have been yet described for *S. sulcata* within this basin. The life cycle of *S. sulcata* has been experimentally studied by Berry and Cannon (3). Larvae hatched from eggs shed with turtles’ feces can infect molluscs through inhalation in the siphon; third stage larvae are generally found in the adductor muscle of the invertebrates. After molting to L4, larvae of about 5 mm length are able to infect sea turtles through ingestion and eggs deposition begins about 6 months after infection.

Even though a low risk for warm-blooded animals has been demonstrated by experimental infections (3), zoonotic potential of *S. sulcata* still has to be completely excluded. Besides, presence of larval stage *S. sulcata* in edible scallops has substantial implications in the depreciation of the product, as well as consequences for health and hygiene requirements following legislation.

Here, we report on the identification of two intermediate hosts of *S. sulcata* in the Adriatic Sea; samples of *S. sulcata* from definitive and intermediate hosts and from different geographical areas have been molecularly characterized and the genomic features have been compared between different developmental stages and marine basins (Adriatic *vs*. Tyrrhenian).

## Materials and Methods

### Morphological Characterization

Intact adult specimens of *S. sulcata* (*n* = 19) were isolated from the stomach and upper part of the intestine of loggerhead turtles stranded along NW Adriatic (*n* = 10) and Tyrrhenian coasts (*n* = 9) of Italy in the period 2012–2015. Adult parasites (*n* = 12), with intact cephalic end and tail, were clarified in Amman's lactophenol solution and subsequently observed at light microscope (Olympus, ACH 40X-2) by NIS-Elements D software (Nikon). Before clarification with Amman's lactophenol, a fragment of all adult specimens was obtained and used for molecular study.

Larvae of nematodes (*n* = 277) were isolated from the adductor muscle of 35 specimens of *Pecten jacobaeus* obtained during regular sanitary inspection by the Sanitary Services of Public Health Authority in Venice province. The specimens belonged to four different production batches, collected between May 2017 and March 2018. Additionally, two similar, non-viable larvae were recovered from two specimens of *Aequipecten opercularis* in May 2019. All specimens of *P. jacobeus* and *A. opercularis* were from Northern Adriatic (FAO zone 37.02.01).

An overall number of 10 larvae, selected from all batches and obtained from both mollusc species were submitted for morphological characterization. Twenty larvae, included the just aforementioned 10 individuals, were included in the molecular study. Morphometric features of adult and larvae have been compared using keys available in literature (3, 20).

Since all turtles were found to be already dead at the time of stranding, no ethical approvals were required for the development of this study.

### Molecular Analyses

DNA was isolated from adult nematodes (*n* = 19) and larvae (*n* = 20) using the extraction kits NucleoSpin^®^ Tissue Kit (Macherey-Nagel, Germany) and the QIAamp DNA Mini Kit (Qiagen), respectively, according to manufacturer's instructions. The entire rDNA fragment comprising ITS1, 5.8S, and ITS2 were amplified using previously described primers NC5 (5′-GTA GGT GAA CCT GCG GAA GGA TCA TT-3′) and NC2 (5′-TTA GTT TCT TTT CCT CCG CT-3′) by polymerase chain reaction (21). The mt-*cox1* and *cox2* genes were amplified as well, with primers JB3 (5′-TTT TTT GGG CAT CCT GAG GTT TAT-3′) and JB4.5 (5′-TAA AGA AAG AAC ATA ATG AAA ATG-3′) (22) and primers 211F (5′-TTT TCT AGT TAT ATA GAT TGR TTY AT-3′) and 210R (5′-CAC CAA CTC TTA AAA TTA TC-3′) (23), respectively.

The PCR for ITS region was performed in a 50 μl reaction, comprising 1 μl DNA, 1.5 mM MgCl_2_, 0.2 mM dNTPs (Thermo Fisher Scientific), 1X PCR buffer, 0.25 μM of each forward and reverse primer, 0.025 U Platinum Taq DNA Polymerase (Invitrogen), with the remainder of the volume made of sterile water. Cycling conditions comprised an initial activation step at 95°C for 2 min., followed by 35 cycles of 95°C for 30 s, 55°C for 30 s, 72°C for 75 s, with a final extension step of 72°C for 10 min.

The PCR for *cox1* region was performed in a 30 μl reaction volume, comprising 1–3 μl DNA, 2.5 mM MgCl_2_, 0.5 mM dNTPs (MBI Fermentas, Germany), 1X PCR buffer, 1.25 μM of each forward and reverse primer, 1U Platinum Taq DNA Polymerase (Invitrogen), with the remainder of the volume made of sterile water. Cycling conditions comprised an initial activation step at 95°C for 2 min., followed by 35 cycles of 94°C for 40 s, 50°C for 30 s, 72°C for 30 s, with a final extension step of 72°C for 5 min.

The PCR for *cox2* region was performed in a 50 μl reaction volume comprising 5 μl DNA, 2.5 mM MgCl_2_, 0.2 mM dNTPs (Thermo Fisher Scientific, USA), 1X PCR buffer, 0.5 μM of each forward and reverse primer, 2U AmpliTaq DNA Polymerase (Invitrogen™, USA), with the remainder of the volume made of sterile water. Cycling conditions comprised an initial activation step at 95°C for 10 min., followed by 50 cycles of 94°C for 30 s, 48°C for 30 s, 72°C for 45 s, with a final extension step of 72°C for 7 min. Negative controls were subjected to amplification together with experimental samples in all PCR assays, as well as a positive control, represented by DNA of *Anisakis simplex* sensu stricto (previously identified and provided by the National Reference Center for Anisakiasis—Istituto Zooprofilattico Sperimentale della Sicilia).

The PCR products were resolved in 1% agarose gel with GelRed^®^ Nucleic Acid Gel Stain 10.000X (Biotium, USA) and SYBR^®^ Safe DNA gel stain (Invitrogen™, USA). The amplicons of PCR (fragments of expected size: 900 bp for ITS, 710 bp and 610 bp for *coxI* and *cox2*, respectively) were directly sequenced by Macrogen (Macrogen Europe, the Netherland) or at the Istituto Zooprofilattico Sperimentale delle Venezie trough the ABI Prism 3130xl Genetic Analyzer (Life Technologies).

The chromatograms were corrected using the software ChromasPro version 2.4.3 (Technelysium Pty Ltd, Australia). The consensus sequences were assembled with the program SeqMan available in the DNAstar package. The consensus sequences were compared with the non-redundant data base available in the GenBank database using the software BLAST (24).

### Sequence Analysis

Obtained sequences were aligned using MAFFTv7.450 (25) and the alignment was visually inspected to evaluate the quality and identify potential misalignments, suggestive of poor quality sequences. Sequences ends were trimmed to obtain a final alignment where a full coverage was present in the whole considered region. An additional alignment obtained concatenating *cox*1, *cox*2 and ITS genes was also created. Raw genetic distances were calculated using the *ape* package in R.

The sequence suitability for phylogenetic analyses was assessed by likelihood mapping analysis, performed using IQ-TREE (26) and phylogenetic tree were reconstructed with the same software, selecting as substitution model the one with the lowest Akaike Information Criterion (AIC), calculated using jModelTest (27). To evaluate if considered genes could be suitable for species identification, a collection of reference sequences of the same genes from individuals belonging to the family Anisakidae was downloaded from Genbank. Obtained sequences were aligned with those obtained in the present study and trimmed to obtain a final alignment where a full coverage was present. To reduce the number of sequences and increase the results interpretability, only one individual was selected as representative of all unique sequences (i.e., 100% percentage of identity). Phylogenetic trees were reconstructed using the previously described approach.

## Results

### Morphological Characterization

#### Adult Nematodes

Adult and immature adult specimens were found free within gastric lumen or fixed with the cephalic end in the stomach wall. Morphological features were in accordance with the description of *S. sulcata* by Berry and Cannon (3) and are here briefly reported, together with measurements (in μm).

Cephalic end with three conspicuous lips and interlabia. Excretory pore observable at the base of the lips. Nerve ring at 828 ± 171 from the apical end. Esophagus, 4,705 ± 1,234 long, terminates in the ventriculum (length 995 ± 542, width 512 ± 215), without any diverticula. An intestinal caecum is present as a small sac-like structure parallel to the ventriculum. Spicules well-visible, cuticularized (1,735 ± 884 long); several pre-cloacal and post-cloacal papillae are visible.

#### Larvae

**Figure 1:**
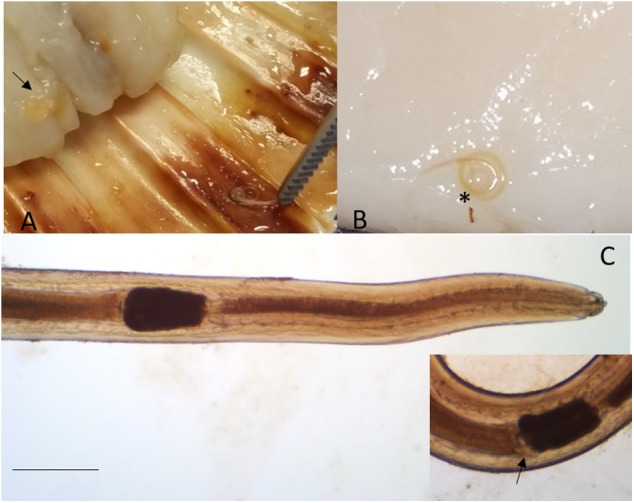
*Sulcascaris sulcata* larvae L4. In **(A)**, larvae (black arrow) are visible within the adductor muscle of the scallop *P. jacobeus*. In **(B)**, enlargement of the dissected muscle with one coiled larva (^*^). In **(C)**, anterior part of L4 showing the ventricle (^*^) and caecum (inset, black arrow) (scale bar: 500 μm).

Larvae collected from the adductor muscle of *P. jacobeus* and *A. opercularis* showed morphological features compatible with stage L4 of *S. sulcata* (Figure 1). More in detail, the larvae, from 9,737 to 21,752 μm long, showed rudimental lips, well-separated from the body, with no ventral tooth; excretory pore was visible at 92.4 ± 33.2 from the extremity, slightly caudal to the lips. A ventriculum (304.4 ± 72 μm long) without lateral diverticula was visible, ending with a rounded fund; intestinal caecum was visible in some specimens as a rudimental sac lateral to the ventriculum, developing cranially. Esophagus 1,522 ± 378 long. Nerve ring at 92 ± 33 from the apical end. A mucron was visible on the tip of the tail.

### Molecular Analyses

Amplification and sequencing of ITS, *cox*1 and *cox*2 regions resulted in 39 good quality consensus sequences for each marker. The BLAST research in GenBank revealed *Contracaecum* spp. to provide the most significant alignment with ITS and *cox*1 sequences from our specimens (86% of alignment with *Contracaecum osculatum* [accession number: MN428821] for ITS, 87–88% of alignment for *cox*1 with *Contracaecum ogmorhini* [AJ616898]), being the sequences of these two DNA fragments absent in Genbank for the species *S. sulcata*. Conversely, *cox*2 sequences blasted with 100% of identity with sequences of *S. sulcata* already present in the database (HQ328505). Newly produced sequences were deposited in GenBank under accession numbers MN712341-MN712379 (ITS), MN713844-MN713882 (*cox*1), and MN713883-MN713921 (*cox*2).

### Phylogenetic Analyses

**Figure 2:**
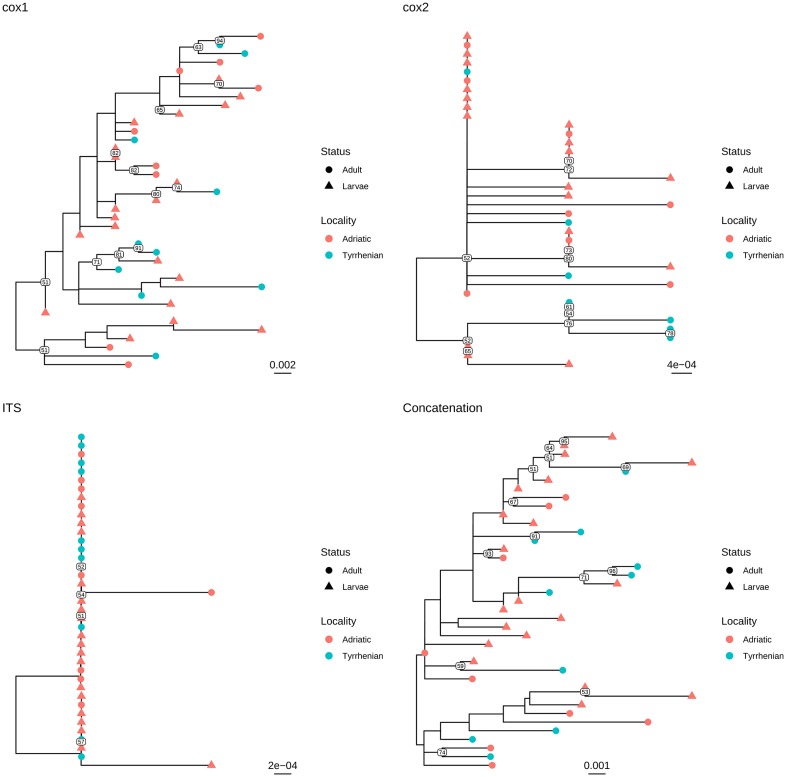
Maximum likelihood phylogenetic tree reconstructed based on the partial sequences of *cox*1, *cox*2, and ITS genes obtained in the present study. A phylogenetic tree was reconstructed also based on the concatenation of the above mentioned genes. The different collection localities have been color coded, while developmental stages have been marked with different tip symbols.

Independently of the considered gene, the collected samples displayed a high genetic similarity (Table 1). No geographic clustering could be identified between sequences obtained from samples collected in the Adriatic or Tyrrhenian Sea (Figure 2), and a clear overlapping in genetic distances within and between basins could be observed (Supplementary Figure 1). Especially, some parasites sampled in the two considered basins showed identical or extremely related sequences (p-distance lower than 0.005), at least in the considered genes.

**Table 1:** Summary statistics of the pairwise genetic distance (p-distance) calculated for *cox*1, *cox*2, ITS and their concatenation.

**Gene**	**Sea**	**Mean**	**Min**	**Max**
*cox1*	All	0.015	0.000	0.030
	Adriatic	0.015	0.000	0.030
	Tyrrhenian	0.016	0.002	0.025
	Adriatic-Tyrrhenian	0.016	0.005	0.030
*cox2*	All	0.004	0.000	0.010
	Adriatic	0.003	0.000	0.008
	Tyrrhenian	0.005	0.000	0.008
	Adriatic-Tyrrhenian	0.005	0.000	0.010
ITS	All	0.0005	0.000	0.005
	Adriatic	0.001	0.000	0.005
	Tyrrhenian	0.001	0.000	0.002
	Adriatic-Tyrrhenian	0.001	0.000	0.003
Concatenation	All	0.006	0.001	0.013
	Adriatic	0.005	0.001	0.011
	Tyrrhenian	0.006	0.002	0.009
	Adriatic-Tyrrhenian	0.006	0.001	0.013

*Values have been calculated both globally and for each Sea. Additionally, the summary statistics of genetic distances among pair of parasites collected in different basins is reported*.

**Figure 3:**
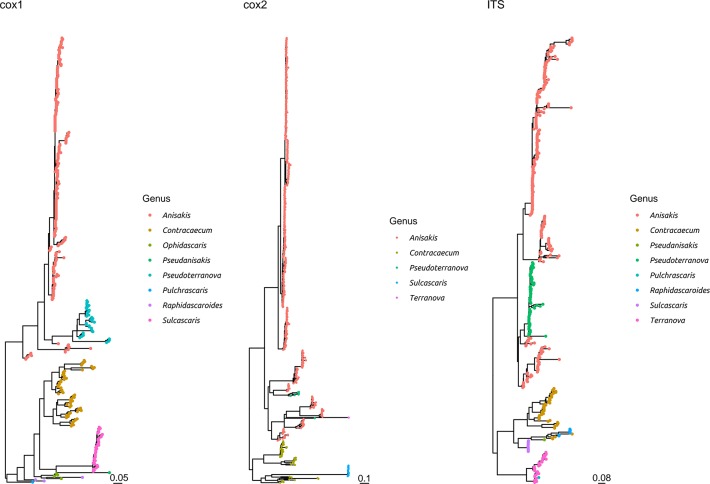
Maximum likelihood phylogenetic tree reconstructed based on the partial sequences of *cox*1, *cox*2, and ITS genes. The sequences obtained in the present study plus a representative dataset of other Anisakidae members have been included in the analysis. The different genera have been color coded.

Similar results were observed when adults and larvae were compared. Identical sequences could be observed also between larvae collected from *Aequipecten* and *Pecten* scallops (Figure 3 and Supplementary Figure 2). Therefore, a common allele pool could be detected in the two basins (with the exception of *cox*1 gene) and in the different developmental stages.

The comparison with other members of the same family demonstrated that *S. sulcata* formed a clear monophyletic cluster, distantly related to other genera, independently from the considered gene (Figure 3 and Supplementary Figure 3).

## Discussion

This study reports the first identification of two intermediate hosts for *S. sulcata* within the Mediterranean basin, both belonging to the family Pectinidae. Larvae of *S. sulcata* have been reported in Australian and Western Atlantic waters from several bivalve species of Pectinidae including *Pecten* spp. (28), *Argopecten gibbus* (18), and *Amusium balloti* (29), and Mactridae including *Spisula solidissima* (2, 30). Additionally, experimental infection was successful in *Isognomon ephippium* (Isognomonidae) and *Pinctada* spp. (Pteriidae) (3) and species of Gastropoda *Cypraea tigris* and *Fasciolaria* sp. are as well included in the list of intermediate hosts of *S. sulcata* (15, 31). In this scenario, we can easily hypothesize that several other intermediate hosts probably exist within the Mediterranean basin for *S. sulcata*. Even though *P*. *jacobeus* and *A*. *opercularis* are common commercial species for human consumption, little likelihood of danger for human health exists after ingestion of *S. sulcata* larvae, being the elective definitive host of this nematode represented by cold-blooded vertebrates. Nevertheless, the anisakid *Hysterothylacium aduncum*, whose definitive hosts are cold-blooded species, is reported in humans as a rare agent of disease (32, 33) and, similarly, larvae of the nematode *Echinocephalus sinensis*, a parasite of elasmobranchs, have been demonstrated to be able to infect primates (34). The habits of cooking scallops before consuming them is protective toward accidental infections, nevertheless, presence of larvae or discolouration of the adductor muscle, due to the presence of the symbiont protozoan *Urosporidium spisuli* (35), justifies the depreciation when not the exclusion of the product from the market when infected by *S. sulcata* larvae.

Infections by *S. sulcata* are described in the Mediterranean population of loggerhead turtles with prevalence ranging around 20% (6, 10, 13), with significantly higher values among loggerhead turtles feeding in Adriatic compared to Tyrrhenian, up to 30% (10). The particular ecological features of the Adriatic basin together with its variety and abundance of suitable preys, make it one of most populated neritic foraging ground for loggerhead turtles within the Mediterranean Sea. Several studies have emphasized the importance of benthic molluscs in loggerhead turtle diet, with large sized species being highly represented and constituting an energetically valuable food item (36, 37). A study by Lazar et al. (19) upon loggerhead turtles feeding in the Northern Adriatic grounds also reported molluscs to be an essential part of sea turtles' diet. Crushed shells of the large-sized bivalves *A. opercularis* and *P. jacobaeus* were identified among the preys in that study, thus we can assume that larvae of *S. sulcata* within the adductor muscle of specimens of the aforementioned bivalve species reasonably can reach the definitive hosts during the turtles' foraging in Northern Adriatic waters. The fidelity of sea turtles to selected foraging grounds indeed promotes completion of the life cycle of the nematode in specific areas, with turtles acting as perpetrators of the parasites life cycle by shedding negatively-buoyant eggs with their feces. Nevertheless, long distance migration of turtles could justify also the expansion of the geographical range of the parasites. This is supported by molecular evidence in this study, which highlights the genetic similarity between parasites isolated in Adriatic and Tyrrhenian waters. In fact, the analysis of three different genes demonstrated the presence of an essentially common allele pool in the two basins, as well as the absence of any geographical clustering among the sequences collected in the Adriatic and Tyrrhenian Seas (Figure 2). Although the limited phylogenetic signal warrants caution in phylogenetic analysis evaluation, the results of individual and concatenated genes analysis suggest a wide circulation of those parasites in the Mediterranean, likely mediated by final host migration. While studies on loggerhead turtles movements within Mediterranean region suggest a tendency to foraging grounds fidelity and shorter range movements, exceptions have been reported, especially with juveniles showing a propensity to wander over quite large areas. Moreover, a clear overlapping is present between typical movements areas of northern and southern Adriatic, Ionian, south-central Mediterranean and Tyrrhenian Seas, which could facilitate parasite transmission and dispersal (38). Unfortunately, the absence of *S. sulcata* sequences collected in other regions hinders a more detailed analysis and further, collaborative, efforts will be necessary to understand the molecular epidemiology and phylogeography of this parasite.

The high genetic homogeneity, especially in the ITS and, to a lesser extent, *cox*2 genes (lower than 1% maximum genetic distance) demonstrates that the investigated genes could be considered an adequate target for species identification. This is confirmed by the comparison with other Anisakidae members, highlighting that *S. sulcata* formed a clearly independent cluster. Nevertheless, a broader collection of *S. sulcata* sequences from individuals sampled all around the world would be of benefit to establish clear species boundaries.

Based on these evidences, the high percentage of identity of the analyzed genes between adults and larvae confirmed the morphological classification of the latter as *S. sulcata* (Figure 2). Molecular methods could thus safely replace morphological methods for larvae identification, coupling a greater easiness of execution and reliability. Additionally, the clear overlapping in genetic distance distribution (Supplementary Figure 2) between strain collected in *Aequipecten* or *Pecten* scallops demonstrates for the first time that this parasite is able to infect also *Aequipecten* species and confirms the broad intermediate host tropism.

## Data Availability Statement

The datasets generated for this study can be found in the GenBank MNMN712341–MN712379, GenBank MN713844–MN713882, GenBank MN713883–MN713921.

## Author Contributions

FM contributed to the conceptualization of the study, coordinated sample collection, performed morphological studies on the parasites and provided funding for the study. FT provided samples, performed molecular analyses and morphological observations on larvae, and revised the paper. GF performed sequences and phylogenetic analyses and drafted the paper. SR and AV provided samples, performed molecular analyses and morphological observations, and revised the paper. MS provided samples and revised the paper. EM performed morphological and molecular analyses and drafted the paper.

### Conflict of Interest

The authors declare that the research was conducted in the absence of any commercial or financial relationships that could be construed as a potential conflict of interest.
