# Supplementary material for: Updates on Ecology and Life Cycle of Sulcascaris sulcata (Nematoda: Anisakidae) in Mediterranean Grounds: Molecular Identification of Larvae Infecting Edible Scallops
Source: Front Vet Sci. 2020 Feb 14;7:64. doi: 10.3389/fvets.2020.00064 (PMC7033499; doi:10.3389/fvets.2020.00064)
Supplement: Supplementary file 1 [file Data_Sheet_1.PDF]

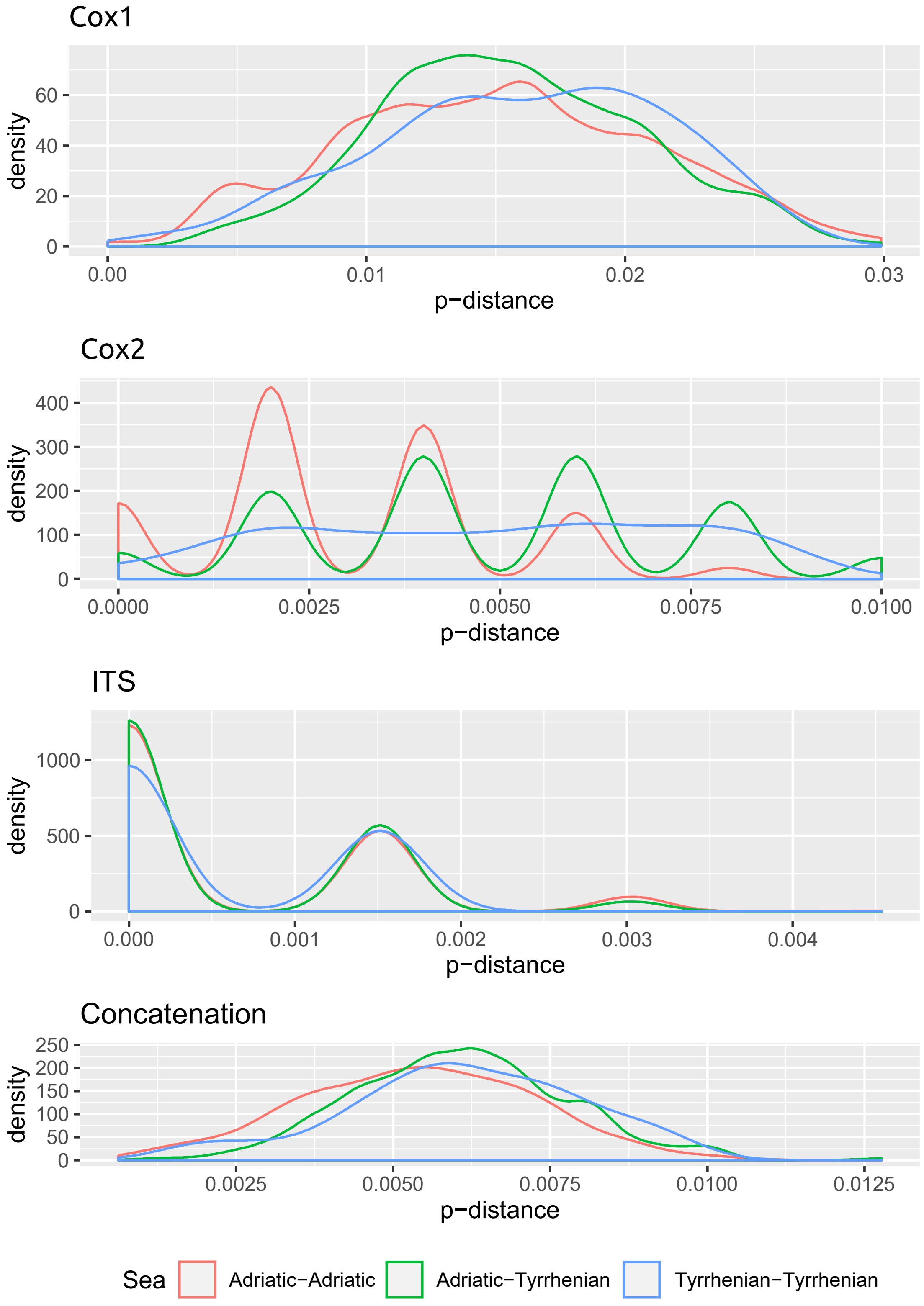

Cox1
density
0
20
40
60
0.00
0.01
0.02
0.03
p−distance
Cox2
density
0
100
200
300
400
0.0000
0.0025
0.0050
0.0075
0.0100
p−distance
ITS
density
0
500
1000
0.000
0.001
0.002
0.003
0.004
p−distance
Concatenation
density
0
50
100
150
200
250
0.0025
0.0050
0.0075
0.0100
0.0125
p−distance
Sea
Adriatic−Adriatic
Adriatic−Tyrrhenian
Tyrrhenian−Tyrrhenian

Supplementary Fig 1. Intra and inter-sea pairwise p-distances among *cox*1, *cox*2, ITS and their concatenation. Density plot reporting the pairwise p-distance distribution within and between seas. Different seas comparisons have been color-coded.
