# Supplementary material for: Updates on Ecology and Life Cycle of Sulcascaris sulcata (Nematoda: Anisakidae) in Mediterranean Grounds: Molecular Identification of Larvae Infecting Edible Scallops
Source: Front Vet Sci. 2020 Feb 14;7:64. doi: 10.3389/fvets.2020.00064 (PMC7033499; doi:10.3389/fvets.2020.00064)
Supplement: Supplementary file 2 [file Data_Sheet_2.PDF]

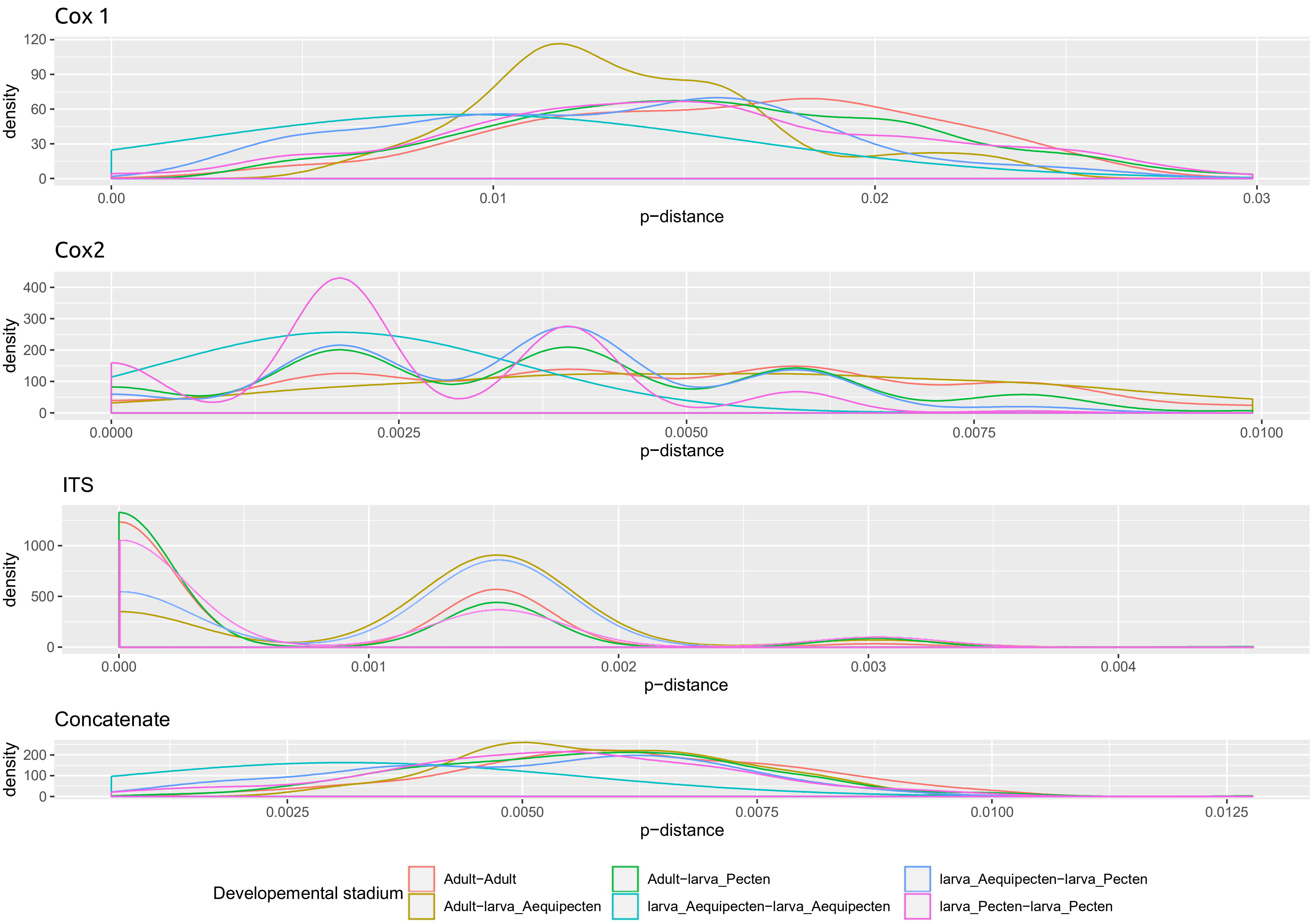

Cox 1
density
p−distance
120
90
60
30
0
0.00
0.01
0.02
0.03
Cox2
density
p−distance
400
300
200
100
0
0.0000
0.0025
0.0050
0.0075
0.0100
ITS
density
p−distance
1000
500
0
0.000
0.001
0.002
0.003
0.004
Concatenate
density
p−distance
200
100
0
0.0025
0.0050
0.0075
0.0100
0.0125
Developemental stadium
Adult−Adult
Adult−larva_Aequipecten
Adult−larva_Pecten
larva_Aequipecten−larva_Aequipecten
larva_Aequipecten−larva_Pecten
larva_Pecten−larva_Pecten

Supplementary Fig 2. Intra and inter-sea pairwise p-distances among *cox*1, *cox*2, ITS and their concatenation. Density plot reporting the pairwise p-distance distribution within and between developmental stages. Different developmental stage comparisons have been color-coded.
