# Supplementary material for: Updates on Ecology and Life Cycle of Sulcascaris sulcata (Nematoda: Anisakidae) in Mediterranean Grounds: Molecular Identification of Larvae Infecting Edible Scallops
Source: Front Vet Sci. 2020 Feb 14;7:64. doi: 10.3389/fvets.2020.00064 (PMC7033499; doi:10.3389/fvets.2020.00064)
Supplement: Supplementary file 3 [file Data_Sheet_3.PDF]

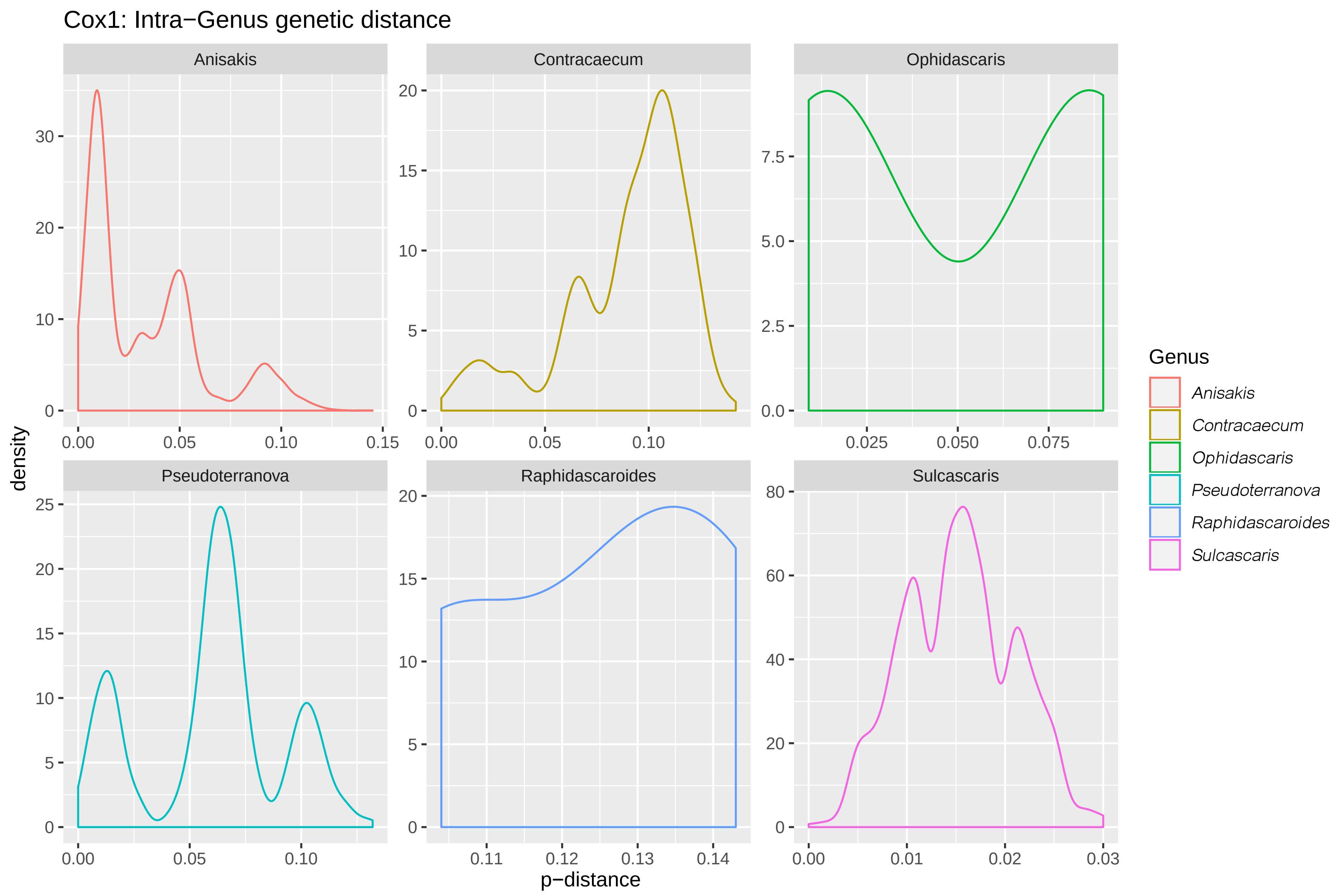
Cox1: Intra−Genus genetic distance
Anisakis
Contracaecum
Ophidascaris
Pseudoterranova
Raphidascaroides
Sulcascaris
density
p−distance
Genus
Anisakis
Contracaecum
Ophidascaris
Pseudoterranova
Raphidascaroides
Sulcascaris

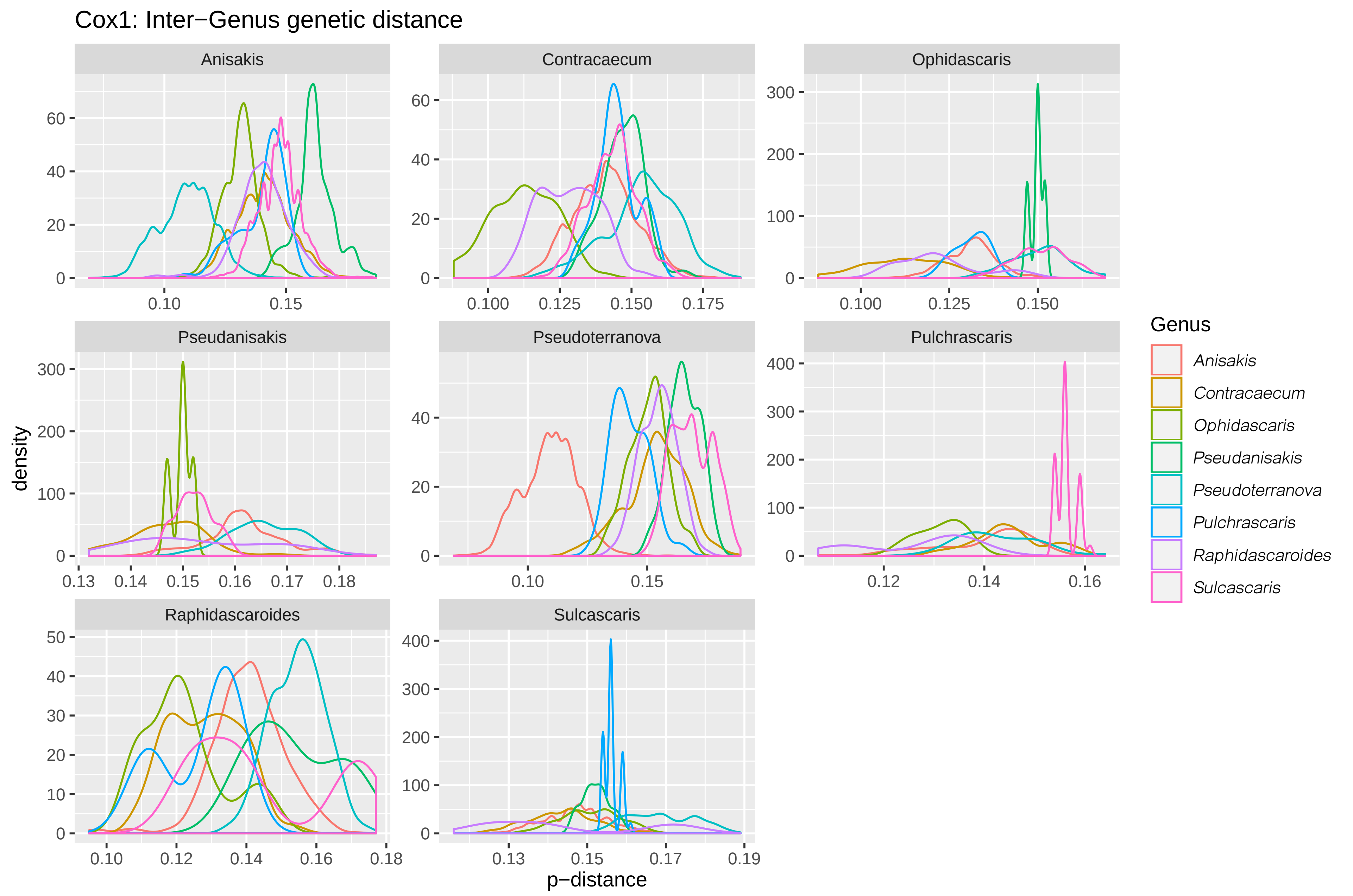
Cox1: Inter−Genus genetic distance
Anisakis
Contracaecum
Ophidascaris
Pseudanisakis
Pseudoterranova
Pulchrascaris
Raphidascaroides
Sulcascaris
density
p−distance
Genus
Anisakis
Contracaecum
Ophidascaris
Pseudanisakis
Pseudoterranova
Pulchrascaris
Raphidascaroides
Sulcascaris

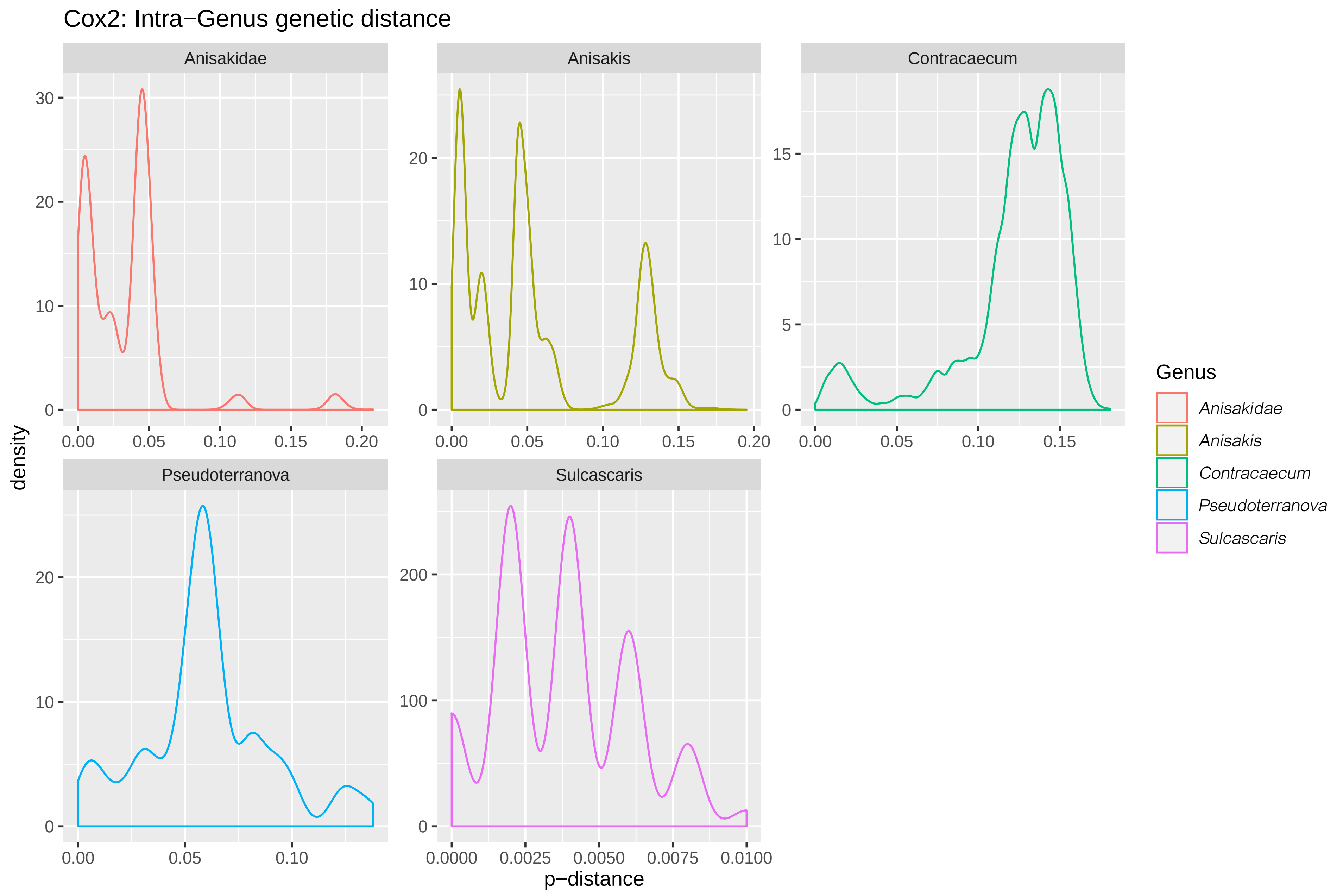
Cox2: Intra−Genus genetic distance
Anisakidae
Anisakis
Contracaecum
Pseudoterranova
Sulcascaris
density
p−distance
Genus
Anisakidae
Anisakis
Contracaecum
Pseudoterranova
Sulcascaris

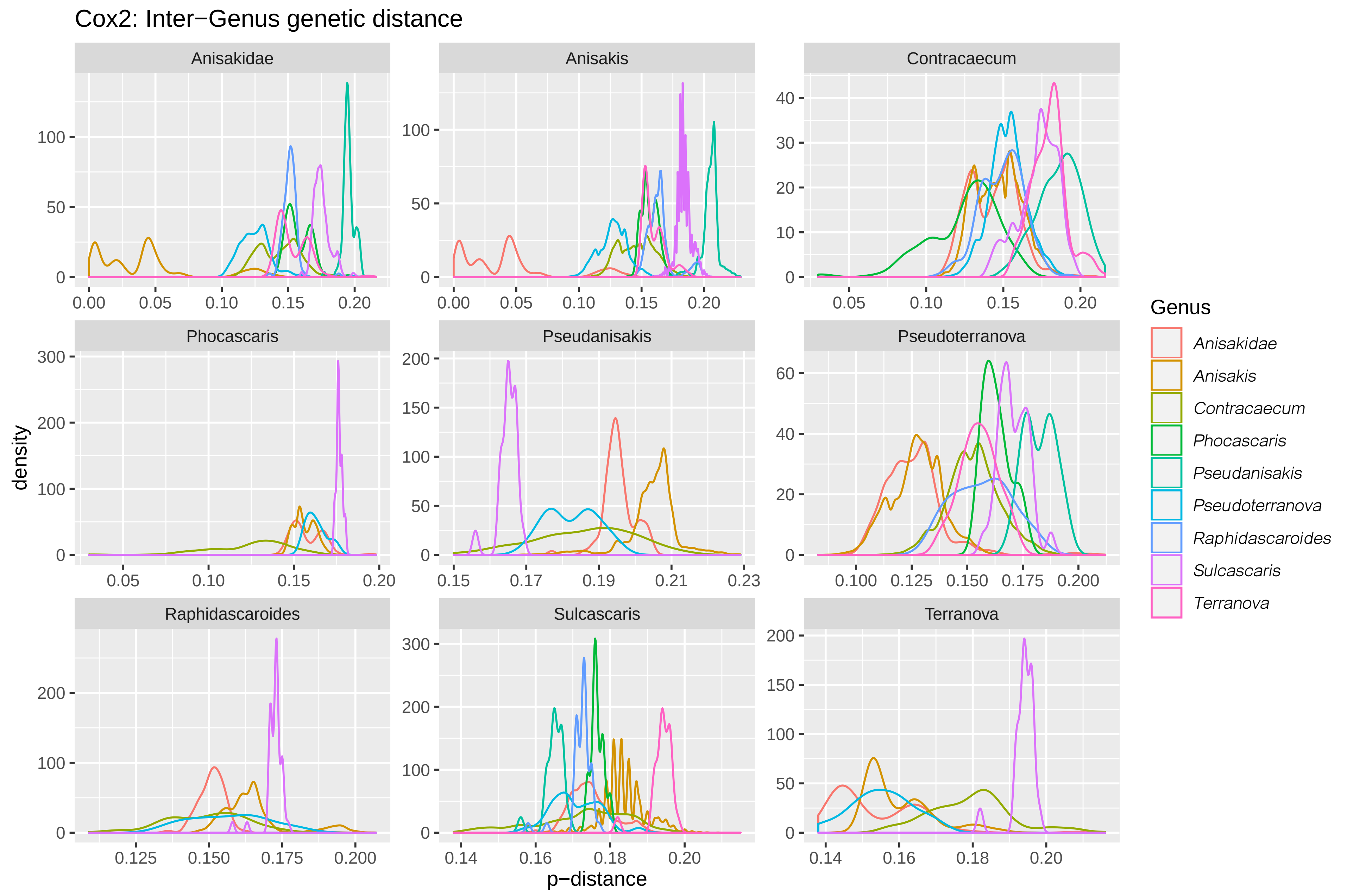
Cox2: Inter−Genus genetic distance
Anisakidae
Anisakis
Contracaecum
Phocascaris
Pseudanisakis
Pseudoterranova
Raphidascaroides
Sulcascaris
Terranova
density
p−distance
Genus
Anisakidae
Anisakis
Contracaecum
Phocascaris
Pseudanisakis
Pseudoterranova
Raphidascaroides
Sulcascaris
Terranova

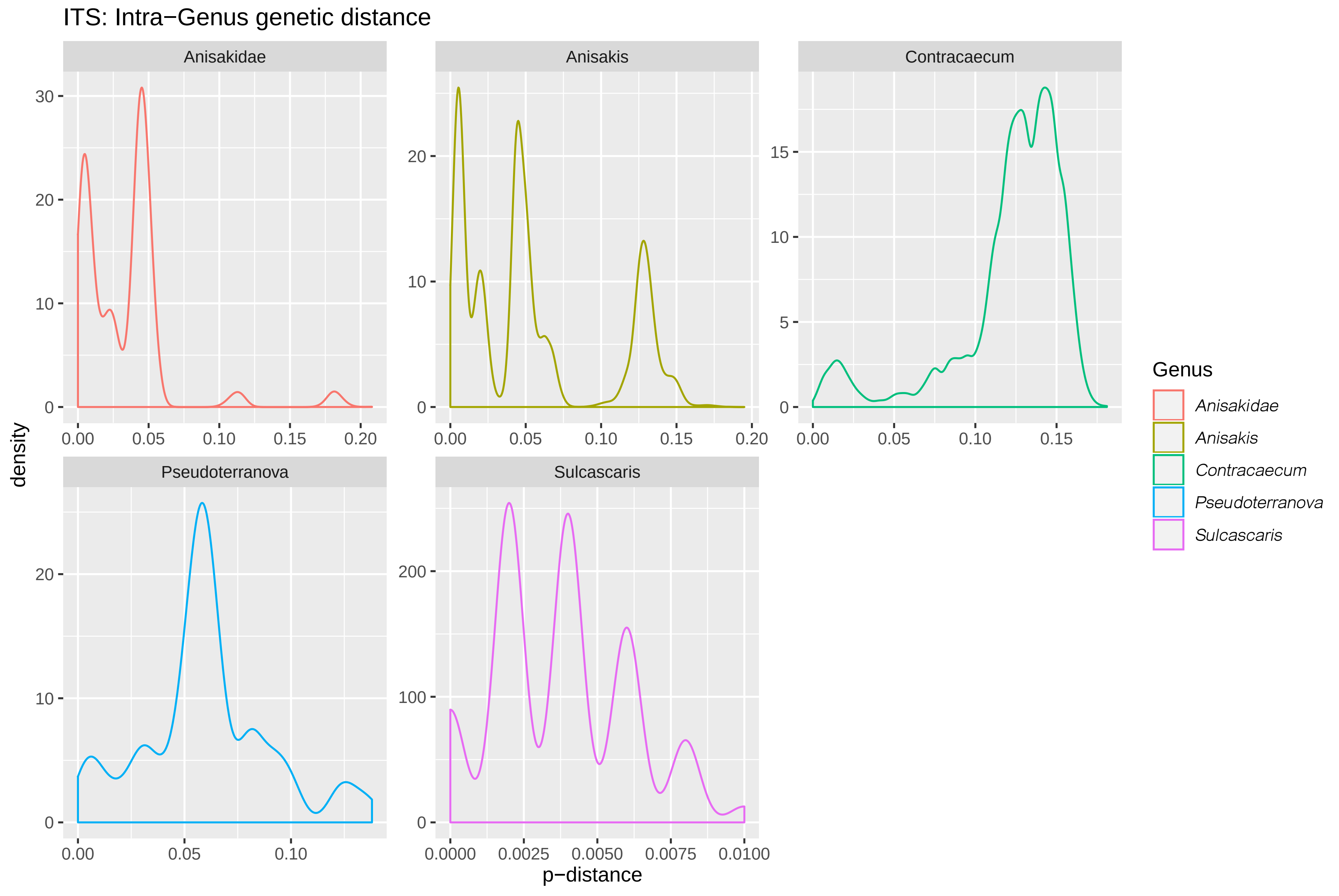
ITS: Intra−Genus genetic distance
Anisakidae
Anisakis
Contracaecum
Pseudoterranova
Sulcascaris
density
p−distance
Genus
Anisakidae
Anisakis
Contracaecum
Pseudoterranova
Sulcascaris

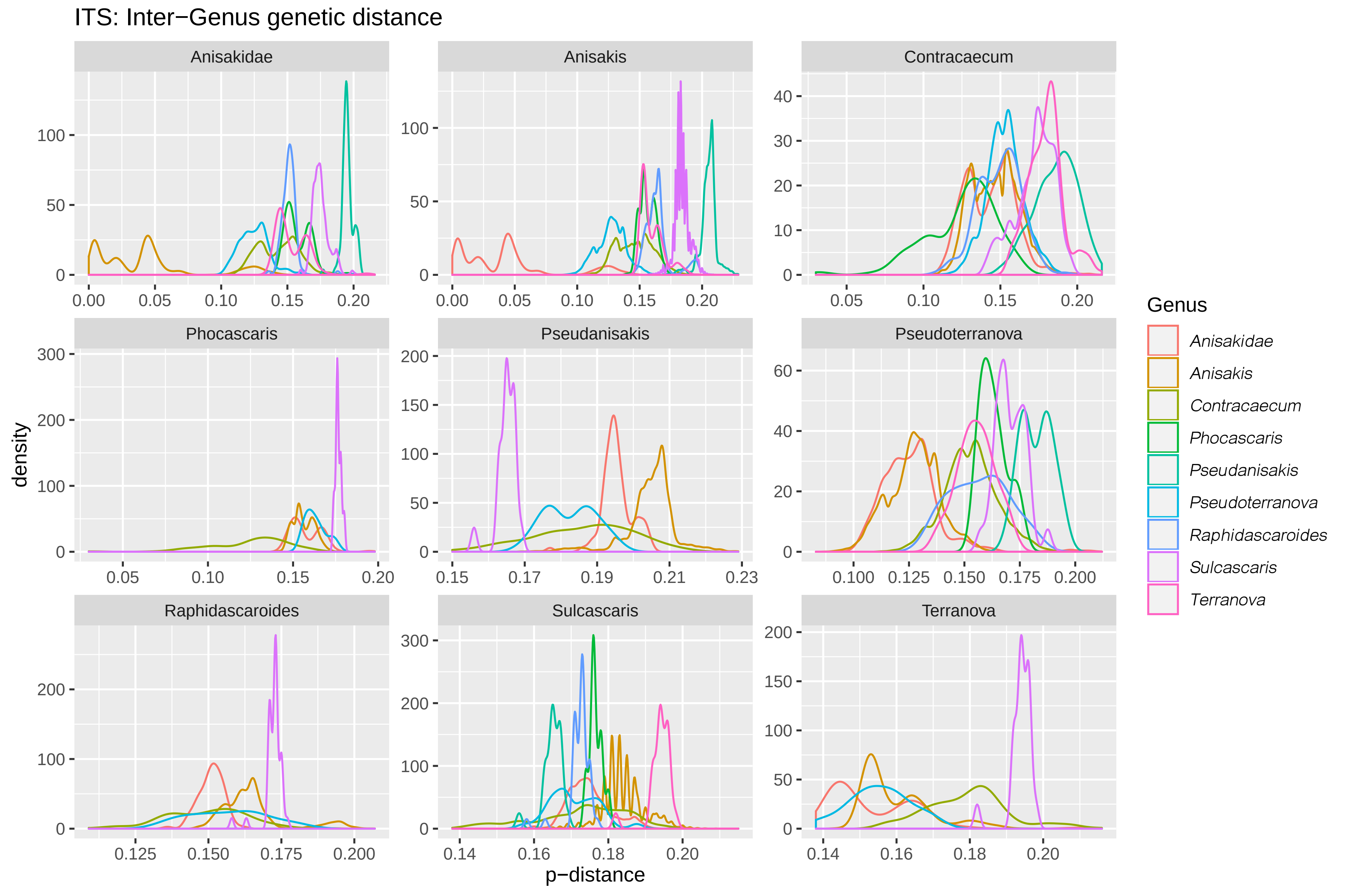
ITS: Inter−Genus genetic distance
Anisakidae
Anisakis
Contracaecum
Phocascaris
Pseudanisakis
Pseudoterranova
Raphidascaroides
Sulcascaris
Terranova
density
p−distance
Genus
Anisakidae
Anisakis
Contracaecum
Phocascaris
Pseudanisakis
Pseudoterranova
Raphidascaroides
Sulcascaris
Terranova

Supplementary Fig 3. Intra (left) and inter-genus (right) pairwise p-distances among *cox*1, *cox*2, and ITS. Density plots reporting the pairwise p-distance distribution within and between genera (color-coded).
